# Supplementary material for: Lineage commitment of embryonic cells involves MEK1-dependent clearance of pluripotency regulator Ventx2
Source: eLife. 2017 Jun 27;6:e21526. doi: 10.7554/eLife.21526 (PMC5487210; doi:10.7554/eLife.21526)
Supplement: Figure 1—figure supplement 1—source data 1. — Details are shown in Figure 1—figure supplement 1 and Materials and methods. DOI: http://dx.doi.org/10.7554/eLife.21526.004 [file elife-21526-fig1-figsupp1-data1.docx]

**Figure 1 -figure supplement1 –source data1**

**Values of blastopore closure ratios.** Details are shown in Figure 1-figure supplement 1 and Materials and Methods.

|  |
| --- |
| \|  \|  \|  \| \| --- \| --- \| --- \| \|  \|  \|  \| \| **TYPE OF EMBRYOS** \| **MEAN BLASTOPORE DIAMETER** \| **BLASTOPORE**  **CLOSURE RATIO** \| \| Ctrl #1 \| 0,085 \| 1,40689655 \| \| Ctrl #2 \| 0,04 \| 0,66206897 \| \| Ctrl #3 \| 0,05 \| 0,82758621 \| \| Ctrl #4 \| 0,11 \| 1,82068966 \| \| Ctrl #5 \| 0,05 \| 0,82758621 \| \| Ctrl #6 \| 0,02 \| 0,33103448 \| \| Ctrl #7 \| 0,01 \| 0,16551724 \| \| Ctrl #8 \| 0,06 \| 0,99310345 \| \| Ctrl #9 \| 0,04 \| 0,66206897 \| \| Ctrl #10 \| 0,065 \| 1,07586207 \| \| Ctrl #11 \| 0,15 \| 2,48275862 \| \| Ctrl #12 \| 0,045 \| 0,74482759 \| \| Ctrl #13 \| 0,05 \| 0,82872928 \| \| Ctrl #14 \| 0,1 \| 1,65745856 \| \| Ctrl #15 \| 0,03 \| 0,49723757 \| \|  \|  \|  \| \| Mk #1 \| 0,305 \| 5,04827586 \| \| Mk #2 \| 0,12 \| 1,9862069 \| \| Mk #3 \| 0,26 \| 4,30344828 \| \| Mk #4 \| 0,155 \| 2,56551724 \| \| Mk #5 \| 0,34 \| 5,62758621 \| \| Mk #6 \| 0,235 \| 3,88965517 \| \| Mk #7 \| 0,125 \| 2,06896552 \| \| Mk #8 \| 0,345 \| 5,71034483 \| \| Mk #9 \| 0,25 \| 4,13793103 \| \| Mk #10 \| 0,19 \| 3,14482759 \| \| Mk #11 \| 0,28 \| 4,63448276 \| \| Mk #12 \| 0,25 \| 4,13793103 \| \| Mk #13 \| 0,19 \| 3,14482759 \| \| Mk #14 \| 0,23 \| 3,80689655 \| \| Mk #15 \| 0,33 \| 5,46206897 \| \| Mk #16 \| 0,19 \| 3,14482759 \| \| Mk #17 \| 0,31 \| 5,13103448 \| \| Mk #18 \| 0,16 \| 2,64827586 \| \|  \|  \|  \| \| Mk-MO #1 \| 0,395 \| 6,53793103 \| \| Mk-MO #2 \| 0,325 \| 5,37931034 \| \| Mk-MO#3 \| 0,39 \| 6,45517241 \| \| Mk-MO #4 \| 0,395 \| 6,53793103 \| \| Mk-MO #5 \| 0,375 \| 6,20689655 \| \| Mk-MO #6 \| 0,305 \| 5,04827586 \| \| Mk-MO #7 \| 0,4 \| 6,62068966 \| \| Mk-MO #8 \| 0,315 \| 5,2137931 \| \| Mk-MO #9 \| 0,325 \| 5,37931034 \| \| Mk-MO #10 \| 0,385 \| 6,37241379 \| \| Mk-MO #11 \| 0,37 \| 6,12413793 \| \| Mk-MO #12 \| 0,38 \| 6,28965517 \| \| Mk-MO #13 \| 0,285 \| 4,71724138 \| \| Mk-MO #14 \| 0,335 \| 5,54482759 \| \| Mk-MO #15 \| 0,425 \| 7,03448276 \| \| Mk-MO #16 \| 0,41 \| 6,7862069 \| \|  \|  \|  \| \| Mk-MO+Mk #1 \| 0,32 \| 5,29655172 \| \| Mk-MO+Mk #2 \| 0,305 \| 5,04827586 \| \| Mk-MO+Mk #3 \| 0,405 \| 6,70344828 \| \| Mk-MO+Mk #4 \| 0,195 \| 3,22758621 \| \| Mk-MO+Mk #5 \| 0,31 \| 5,13103448 \| \| Mk-MO+Mk #6 \| 0,285 \| 4,71724138 \| \| Mk-MO+Mk #7 \| 0,015 \| 0,24827586 \| \| Mk-MO+Mk #8 \| 0,19 \| 3,14482759 \| \| Mk-MO+Mk #9 \| 0,37 \| 6,12413793 \| \| Mk-MO+Mk #10 \| 0,275 \| 4,55172414 \| \| Mk-MO+Mk #11 \| 0,12 \| 1,9862069 \| \| Mk-MO+Mk #12 \| 0,145 \| 2,4 \| \| Mk-MO+Mk #13 \| 0,255 \| 4,22068966 \| \| Mk-MO+Mk #14 \| 0,305 \| 5,04827586 \| \| Mk-MO+Mk #15 \| 0,43 \| 7,11724138 \| \| Mk-MO+Mk #16 \| 0,435 \| 7,2 \| \| Mk-MO+Mk #17 \| 0,33 \| 5,46206897 \| \| Mk-MO+Mk #18 \| 0,26 \| 4,30344828 \| \| Mk-MO+Mk #19 \| 0,27 \| 4,46896552 \| \| Mk-MO+Mk #20 \| 0,3 \| 4,96551724 \| \| Mk-MO+Mk #21 \| 0,44 \| 7,28275862 \| \| Mk-MO+Mk #22 \| 0,165 \| 2,73103448 \| \| Mk-MO+Mk #23 \| 0,13 \| 2,15172414 \| \| Mk-MO+Mk #24 \| 0,2 \| 3,31034483 \| \| Mk-MO+Mk #25 \| 0,46 \| 7,6137931 \| \| Mk-MO+Mk #26 \| 0,185 \| 3,06206897 \| \| Mk-MO+Mk #27 \| 0,06 \| 0,99310345 \| \| Mk-MO+Mk #28 \| 0,275 \| 4,55172414 \| \| Mk-MO+Mk #29 \| 0,155 \| 2,56551724 \| \| Mk-MO+Mk #30 \| 0,215 \| 3,55862069 \| \| Mk-MO+Mk #31 \| 0,18 \| 2,97931034 \| \| Mk-MO+Mk #32 \| 0,19 \| 3,14482759 \| \| Mk-MO+Mk #33 \| 0,2 \| 3,31034483 \| \| Mk-MO+Mk #34 \| 0,05 \| 0,82758621 \| \| Mk-MO+Mk #35 \| 0,135 \| 2,23448276 \| \| Mk-MO+Mk #36 \| 0,115 \| 1,90344828 \| \| Mk-MO+Mk #37 \| 0,01 \| 0,16551724 \| \| Mk-MO+Mk #38 \| 0,075 \| 1,24137931 \| |
