# Supplementary material for: Lineage commitment of embryonic cells involves MEK1-dependent clearance of pluripotency regulator Ventx2
Source: eLife. 2017 Jun 27;6:e21526. doi: 10.7554/eLife.21526 (PMC5487210; doi:10.7554/eLife.21526)
Supplement: Figure 3—source data 1. — Each value corresponds to the ratio calculated from one individual confocal slice between α and β daughter nuclei (see legend to Figure 3 and Materials and methods for further details). DOI: http://dx.doi.org/10.7554/eLife.21526.010 [file elife-21526-fig3-data1.docx]

**Figure 3-source data 1. Myc signal intensity ratios between daughter nuclei**. Each value corresponds to the ratio calculated from one individual confocal slice between α and β daughter nuclei (see legend to Figure 3 and Materials and Methods for further details).

| **Ventx2-Myc** | **Ventx2-Myc + Mk-MO** | **2SAVentx2-Myc** |
| --- | --- | --- |
| \| 4,513323 \| \| --- \| \| 5,520392 \| \| 4,684906 \| \| 3,579249 \| \| 4,513323 \| \| 5,520392 \| \| 4,684906 \| \| 3,579249 \| \| 2,758953 \| \| 2,164713 \| \| 5,885196 \| \| 4,959427 \| \| 3,809084 \| \| 3,292643 \| \| 2,679929 \| \| 2,235378 \| \| 10,98734 \| \| 9,464646 \| \| 10,13372 \| \| 11,03496 \| \| 9,895197 \| \| 10,04369 \| \| 8,014574 \| \| 7,579808 \| \| 6,592783 \| \| 7,820276 \| \| 6,109925 \| \| 1,989117 \| \| 3,790761 \| \| 13,13739 \| \| 13,13739 \| \| 12,28722 \| \| 13,1332 \| \| 6,532143 \| \| 11,53061 \| \| 10,05634 \| \| 7,549505 \| \| 6,91603 \| \| 2,628853 \| \| 2,320847 \| \| 2,034351 \| \| 1,964912 \| | \| 1,4038 \| \| --- \| \| 1,171756 \| \| 0,8503876 \| \| 0,9158654 \| \| 1,4038 \| \| 1,171756 \| \| 0,8503876 \| \| 0,9158654 \| \| 0,8482853 \| \| 0,8869534 \| \| 0,9465297 \| \| 0,9681404 \| \| 1,227425 \| \| 1,227425 \| \| 0,7252747 \| \| 1,023256 \| \| 1,478666 \| \| 1,60871 \| \| 1,274744 \| \| 1,284329 \| \| 1,113645 \| \| 1,040121 \| \| 0,8955169 \| \| 0,7072098 \| \| 0,5573111 \| \| 0,5650253 \| \| 0,7432678 \| \| 0,8655826 \| \| 0,6982959 \| \| 1,446489 \| \| 1,697961 \| \| 1,598311 \| \| 1,544441 \| \| 1,673422 \| \| 1,592472 \| \| 1,578519 \| \| 1,361912 \| \| 1,098908 \| \| 0,9984125 \| \| 0,8742797 \| \| 0,7545185 \| \| 1,494922 \| \| 1,393574 \| \| 1,232823 \| \| 1,080929 \| \| 0,9904447 \| \| 0,9270497 \| \| 0,8670107 \| \| 0,7549375 \| \| 0,706937 \| \| 0,6726325 \| | \| 2,527126 \| \| --- \| \| 2,293698 \| \| 1,599286 \| \| 1,078452 \| \| 2,039335 \| \| 4,447485 \| \| 3,700821 \| \| 3,419384 \| \| 3,248032 \| \| 2,964486 \| \| 2,790954 \| \| 2,54915 \| \| 2,335131 \| \| 2,180394 \| \| 2,040924 \| \| 0,892921 \| \| 0,6275755 \| \| 0,6144601 \| \| 0,5941537 \| \| 0,7998159 \| \| 0,7568612 \| \| 0,8607703 \| \| 0,9132534 \| \| 1,033708 \| \| 1,170002 \| \| 1,314266 \| \| 1,449759 \| \| 1,606959 \| \| 1,709672 \| \| 1,856514 \| \| 1,88659 \| \| 5,688225 \| \| 2,716838 \| \| 2,093272 \| \| 1,813109 \| \| 1,623057 \| \| 1,502836 \| \| 1,458045 \| \| 1,375822 \| \| 1,294519 \| \| 1,125282 \| \| 1,046801 \| \| 0,9270005 \| \| 1,304573 \| \| 1,643597 \| \| 1,963664 \| \| 2,16672 \| \| 2,76629 \| \| 2,297538 \| \| 2,039766 \| \| 1,990933 \| \| 2,029041 \| \| 1,825285 \| \| 1,949957 \| \| 3,104404 \| |
