# Supplementary material for: Lineage commitment of embryonic cells involves MEK1-dependent clearance of pluripotency regulator Ventx2
Source: eLife. 2017 Jun 27;6:e21526. doi: 10.7554/eLife.21526 (PMC5487210; doi:10.7554/eLife.21526)
Supplement: Supplementary file 1. — (A) Four-cell embryos were injected with 300 pg DN-GSK3 RNA per blastomere. Embryos were collected at stage 25 and processed for WISH analysis with sox2 probe. DN-GSK3 efficiently induced secondary body axes, indicating that the dose used was functional. (B) Embryos injected as in (A), were collected at stage 10.5 and processed for RT-qPCR. (C-D) Embryos injected as in (A) were processed for WISH analysis at early gastrula stage 10.5 with gsc (C, vegetal view) and ventx2 (D, top: vegetal view, bottom: animal view) probes. (E) Embryos injected at the 8 cell stage with 300 pg DN-GSK3 RNA in one dorsal animal blastomere were processed for WISH analysis at late gastrula stage 13 with pou5f3.2 and ventx2 probes (anterior view). (F-G) Four-cell embryos were injected in each cell with 50 pg GFP-CAAX, 50 pg Ventx2-Myc, and 25 ng Mk-MO (F), or 50 pg GFP-CAAX, 50 pg Ventx2-Myc and 300 pg of DN-GSK3 (G) Animal caps were explanted at blastula stage nine and cultured until gastrula stage 11, fixed and processed for anti-Myc (red), and anti-GFP (green) immunostaining, and DNA was stained with DAPI (blue). Note that Ventx2-Myc is detectable only in MEK1 depleted caps. For the qPCR graph, error bars represent s.e.m. values of three independent experiments with two technical duplicates. For statistical analyses, samples were compared with the respective control by Unpaired Student’s t-test. *p<0.05, **p<0.005. In C, D and E, the number of embryos exemplified by the photograph over the total number of embryos analyzed is indicated. In F and G scale bar is 20 μm. DOI: http://dx.doi.org/10.7554/eLife.21526.014 [file elife-21526-supp1.pdf]

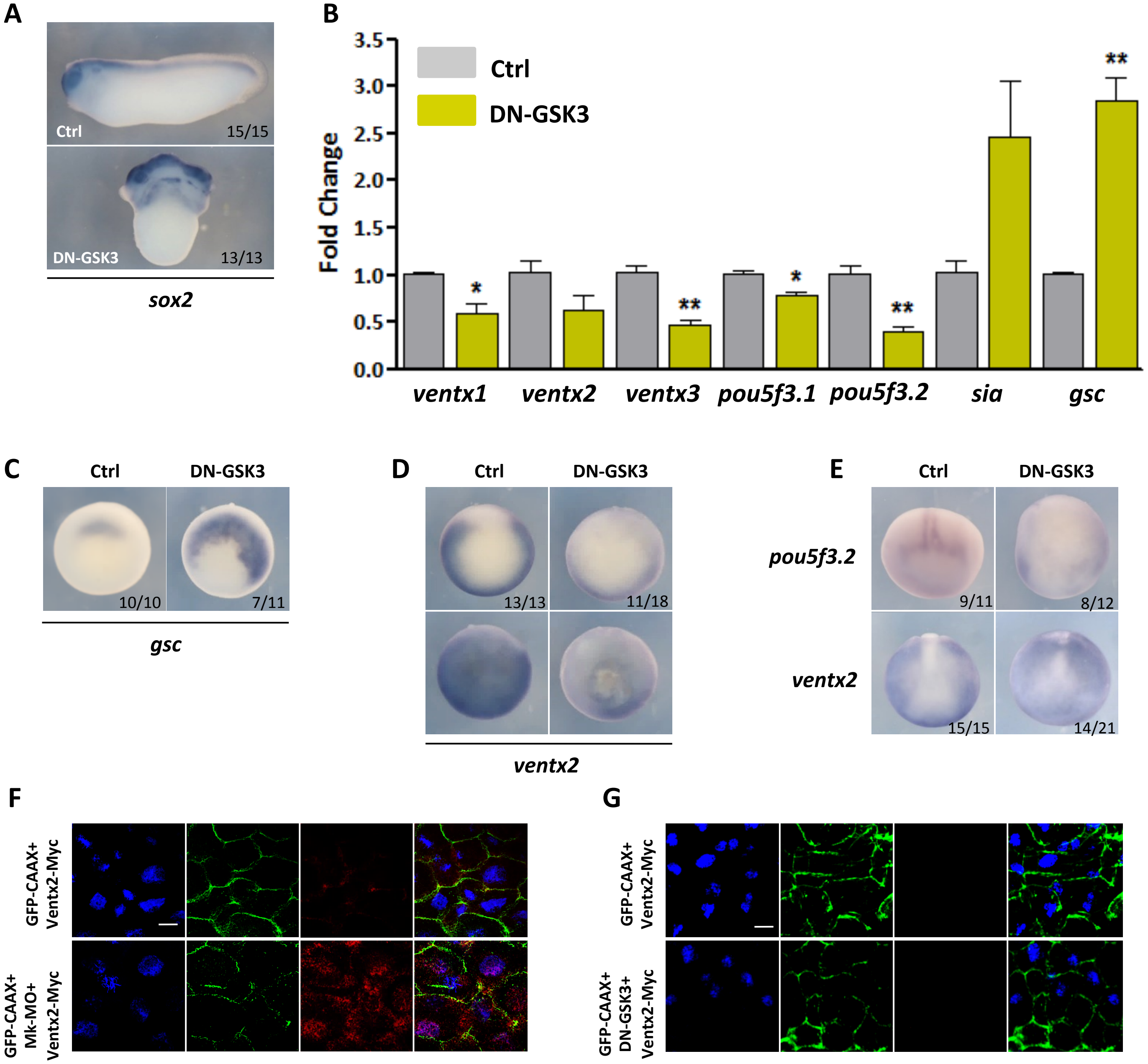

Supplementary file 1. GSK3 is not a negative regulator of the pluripotency gene network and is not required for MEK1-dependent Ventx2 clearance *in vivo*.

A. 4-cell embryos were injected with 300pg DN-GSK3 RNA per blastomere. Embryos were collected at stage 25 and processed for WISH analysis with *sox2* probe. DN-GSK3 efficiently induced secondary body axes, indicating that the dose used was functional. **B.** Embryos injected as in (A), were collected at stage 10.5 and processed for RT-qPCR. **C-D.** Embryos injected as in (A) were processed for WISH analysis at early gastrula stage 10.5 with *gsc* (C, vegetal view) and *ventx2* (D, top: vegetal view, bottom: animal view) probes. **E.** Embryos injected at the 8-cell stage with 300pg DN-GSK3 RNA in one dorsal animal blastomere were processed for WISH analysis at late gastrula stage 13 with *pou5f3.2* and *ventx2* probes (anterior view). **F-G.** 4-cell embryos were injected in each cell with 50pg GFP-CAAX, 50pg Ventx2-Myc, and 25ng Mk-MO (F), or 50pg GFP-CAAX, 50pg Ventx2-Myc and 300pg of DN-GSK3 (G). Animal caps were explanted at blastula stage 9 and cultured until gastrula stage 11, fixed and processed for anti-Myc (red), and anti-GFP (green) immunostaining, and DNA was stained with DAPI (blue). Note that Ventx2-Myc is detectable only in MEK1 depleted caps. For the qPCR graph, error bars represent s.e.m. values of three independent experiments with two technical duplicates. For statistical analyses, samples were compared with the respective control by Unpaired Student's t-test. *P< 0.05, **P< 0.005. In C, D and E, the number of embryos exemplified by the photograph over the total number of embryos analyzed is indicated. In F and G scale bar is 20 μm.
