# Supplementary material for: Lineage commitment of embryonic cells involves MEK1-dependent clearance of pluripotency regulator Ventx2
Source: eLife. 2017 Jun 27;6:e21526. doi: 10.7554/eLife.21526 (PMC5487210; doi:10.7554/eLife.21526)
Supplement: Supplementary file 2. — Boxes indicate members with orthology relationship, like coelacanth Ventx, Xenopus Ventx2 and human VENTX (blue arrows). Sequences were collected from ENSEMBL, JGI, A-STAR and NCBI public databases (see Supplementary file 4). Ventx homeodomain sequences were aligned using Jalview software (RRID:SCR_006459) and the phylogenetic tree was obtained by Neighbor Joining analysis of percentage identity. DOI: http://dx.doi.org/10.7554/eLife.21526.015 [file elife-21526-supp2.pdf]

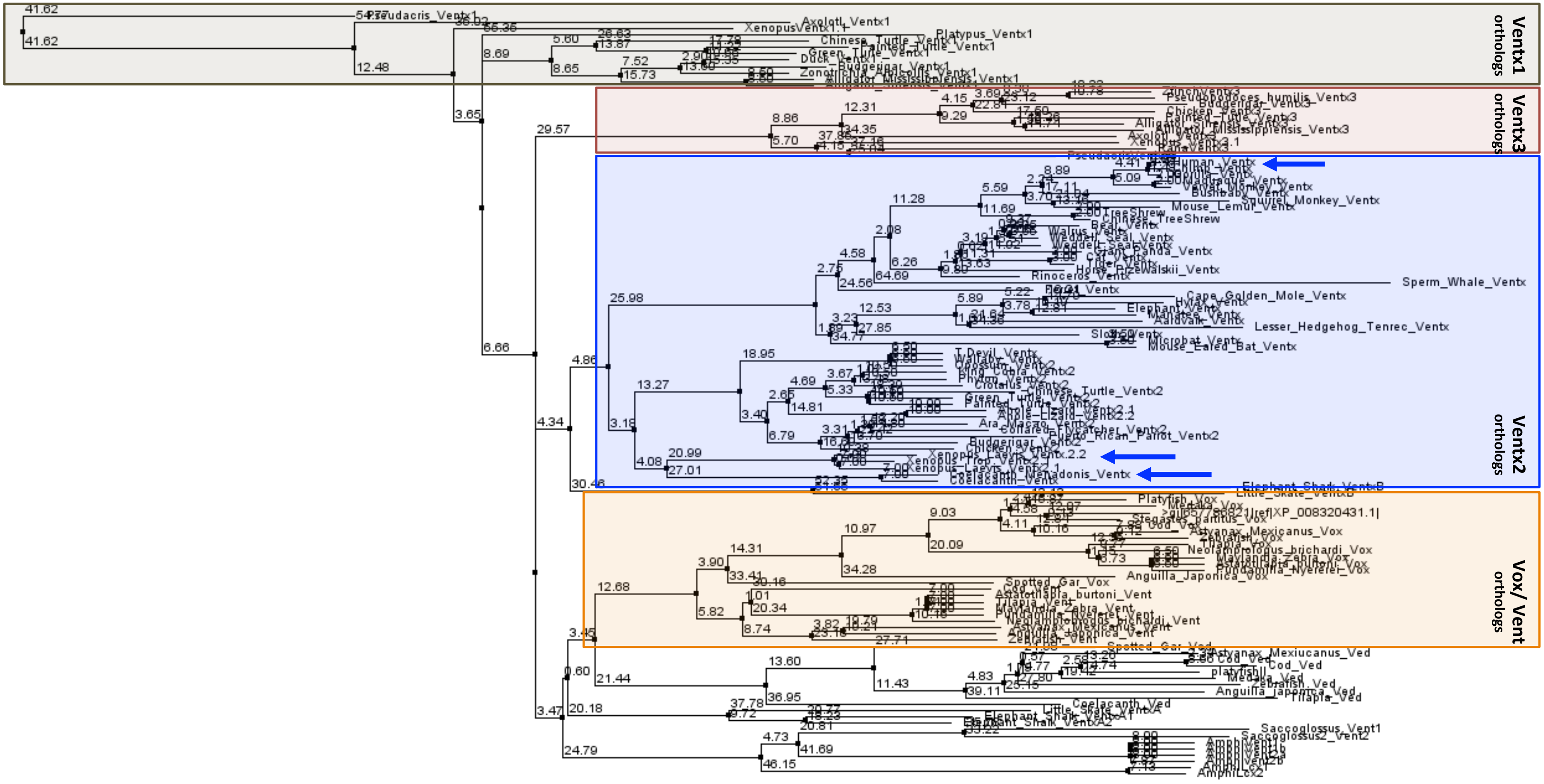

Supplementary file 2. Phylogenetic tree of *Ventx* deuterostome genes.
Boxes indicate members with orthology relationship, like coelacanth *Ventx*, *Xenopus Ventx2* and human *VENTX* (blue arrows). Sequences were collected from ENSEMBL, JGI, A-STAR and NCBI public databases (see Supplementary file 4). Ventx homeodomain sequences were aligned using Jalview software (RRID:SCR_006459) and the phylogenetic tree was obtained by Neighbor Joining analysis of percentage identity.
