# Supplementary material for: Lineage commitment of embryonic cells involves MEK1-dependent clearance of pluripotency regulator Ventx2
Source: eLife. 2017 Jun 27;6:e21526. doi: 10.7554/eLife.21526 (PMC5487210; doi:10.7554/eLife.21526)
Supplement: Supplementary file 3. — (A) Synteny of the Ventx genomic region in gnathostomes. Blue dotted boxes indicate species-specific gene duplication events. Note that a triplication event, giving rise to Ventx1, Ventx2 and Ventx3, occurred in the last common ancestor of tetrapods. One or more Ventx paralogs was subsequently lost during squamata, archosaura and testudina evolution. Mammals lost both Ventx1 and Ventx3 paralogs and exclusively kept Ventx2. Mouse represents an extreme case with a total loss of Ventx genes. (B) Simplified tree of vertebrates, which displays typical situations regarding the number of Ventx genes in main evolutionary branches. DOI: http://dx.doi.org/10.7554/eLife.21526.016 [file elife-21526-supp3.pdf]

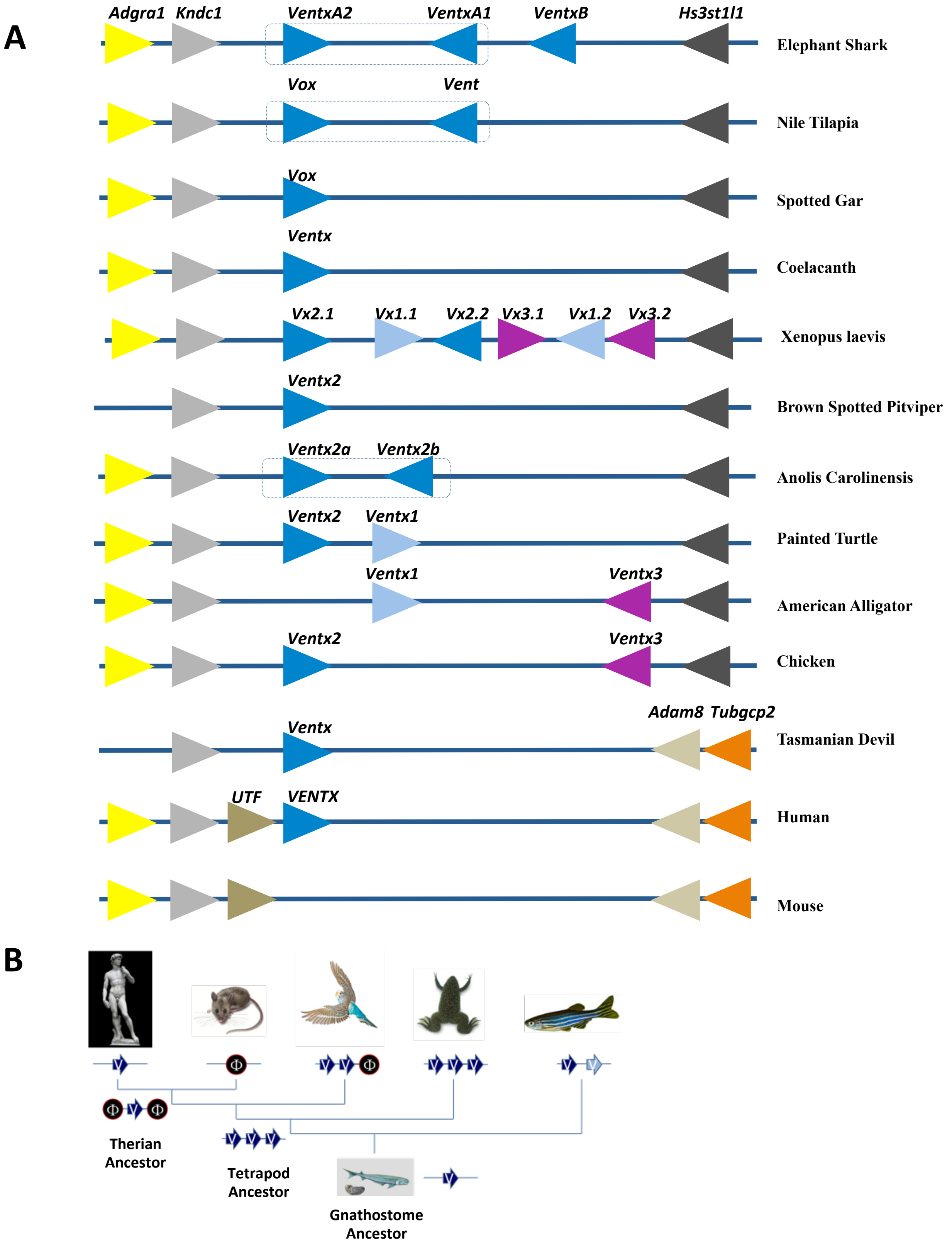

Supplementary file 3. The Evolutionary history of Ventx family genes.
A. Synteny of the *Ventx* genomic region in gnathostomes. Blue dotted boxes indicate species-specific gene duplication events. Note that a triplication event, giving rise to *Ventx1*, *Ventx2* and *Ventx3*, occurred in the last common ancestor of tetrapods. One or more *Ventx* paralogs was subsequently lost during squamata, archosaura and testudina evolution. Mammals lost both *Ventx1* and *Ventx3* paralogs and exclusively kept *Ventx2*. Mouse represents an extreme case with a total loss of *Ventx* genes. **B**. Simplified tree of vertebrates, which displays typical situations regarding the number of *Ventx* genes in main evolutionary branches.
