# Supplementary material for: Lineage commitment of embryonic cells involves MEK1-dependent clearance of pluripotency regulator Ventx2
Source: eLife. 2017 Jun 27;6:e21526. doi: 10.7554/eLife.21526 (PMC5487210; doi:10.7554/eLife.21526)
Supplement: Source data 1. — VENTX homeodomain sequences. DOI: http://dx.doi.org/10.7554/eLife.21526.020 [file elife-21526-data1.docx]

**Source data related to Supplementary file 2: VENTX homeodomain sequences**

>Human_Ventx/92-152 Ventx

PRVRTAFTMEQVRTLEGVFQHHQYLSPLERKRLAREMQLSEVQIKTWFQNRRMKHKRQMQD

>Squirrel_Monkey_Ventx/92-152

PRICTAFSTEQVRALEGVFRHHRYLGPLERKRLAREMQLSELQVKTWFQNRRMKHKQEVQD

>Chimp_Ventx/92-152 Ventx

PRVRTAFTMEQVRTLEGVFQHHQYLSPLERKRLAREMQLSEVQIKTWFQNRRMKHKRQMQD

>Maquaque_Ventx/92-152 Ventx

PRVRTAFTTEQVRTLEGVFQHHQYLSPLERKRLAREMQLSEVQIKTWFQNRRMKHKRQMQE

>Gorilla_Ventx/92-152 Ventx

PRVRTAFTMEQVRTLEGVFQHHQYLSPLERKRLAREMQLSEVQIKTWFQNRRMKHKRQMQD

>Mouse_Lemur_Ventx/85-145 Ventx Microcebus griseorufus]

PRVRTAFSTEQLRALEGVFRHHQYLGPLERKKLAREMRLSEVQIKTWFQNRRMKHKRQMQD

>Bushbaby_Ventx/95-155 Otolemur Garnettii

PRVRTAFSAEQLRALEGVFQHHQYLSPLERKKLAKEMQLSEVQIKTWFQNRRMKHKRQIQD

>Cape_Golden_Mole_Ventx/77-137 Chrysochloris asiatica contig183193

PRVRTAFTAAQVRTLESAFQLHQYLGPQERKKLAKEMCLTEVQIKTWFQNRRMRHKRQMQD

>Hyrax_Ventx/90-150 Ventx

PRVRTAFTAAQVSTLESAFKLRQYLGPQERKKLAREMCLTEVQIKTWFQNRRMKHKRQVQD

>TreeShrew/90-150 Ventx

PRVRTAFTVEQVSALEGAFQHHQYLGPLERKKLAKEMQLSEVQIKTWFQNRRMKHKRQMQD

>Chinese_TreeShrew/132-192 Homeobox protein VENTX [Tupaia chinensis] gi|444726785|gb|ELW67305.1|

PRVRTAFTVEQVSALEGAFQHHQYLGPLERKKLAKEMQLSEVQIKTWFQNRRMKHKRQMQD

>Bear_Ventx/90-150 Ursus maritimus contig2696, whole genome shotgun sequence GenBank: AVOR01002696.1

PRVRTAFTAEQVSTLESAFQHHRYLGPLERGRLAREMRLSEVQIKTWFQNRRMKHKRQLQD

>Microbat_Ventx/91-151 Ventx

PRVRTAFTEEQVSTLESSFQLHRYLDPQERRRLAQTMGLSEVQIKTWFQNRRMKHKRQLQD

>Mouse_Eared_Bat_Ventx/91-151 Homeobox protein VENTX [Myotis davidii] gi|432115368|gb|ELK36785.1|

PRVRTAFTEEQVSTLESSFQLHRYLDPQERRRLAQTMGLSEVQIKTWFQNRRMKHKRQLQD

>Horse_Przewalskii_Ventx/91-151 XP_008523846

PRVRTAFTVEQVSTLESSFQHRRYLGPLERRRLAREMRLSEVQIKTWFQNRRMKHKRQLQD

>Rinoceros_Ventx/91-151

PRVRTAFTSEQVSTLESSFQHHRYLGPLERRRLAREMQLSEVQIKTWFQNRRMKHKRQLQD

>Walrus_Ventx/89-149

PRVRTAFTAEQVSTLESAFQHHRYLGPLERRRLAREMRLSEVQIKTWFQNRRMKHKRQLQD

>Ferret_Ventx/89-149

PRVRTAFSAEQVSTLESAFQRRRYLGPAERRRLARDMRLSEVQIKTWFQNRRMKHKRQLQD

>Cat_Ventx/90-150

PRVRTAFTAEQVSTLESAFQHQRYLGPLERRKLAREMRLSEVQIKTWFQNRRMKHKHQLQD

>Tiger_Ventx/90-150

PRVRTAFTAEQVSTLESAFQHQRYLGPLERRKLAREMRLSEVQIKTWFQNRRMKHKHQLQD

>Aardvark_Ventx/90-150 Orycteropus afer afer contig064492

PRARTAFTTVQISTLESAFQLHQYLGPQERKKLARQMELTEVQIKTWFQNRRMKQKRQMQD

>Vervet_Monkey_Ventx/87-147 Scaffold KE147988.1: 1,807-4,222 ENSP00000357556

PRVRTAFTTEQVRTLEGVFQHHQYLSPLERKRLAREMQLSEVQIKTWFQNRRMKHKRQMQE

>Elephant_Ventx/91-151

PRVRTAFTTAQISTLESAFKLHQYLGPQERKKLAKEMHLTEVQIKTWFQNRRMKHKRQLQD

>Giant_Panda_Ventx/88-148

PRVRTAFTAEQVSTLESAFQHHRYLGPLERRTLAREMRLSEAQIKTWFQNRRMKHKRQLQD

>Weddell_Seal_Ventx/89-149

PRVRTAFTAEQVSTLESAFQHHRYLGPLERRRLAREMRLSEVQVKTWFQNRRMKHKRQLQD

>T.Devil_Ventx/126-186 Ventx

RRLRTAFTTKQISTLESSFKRHHYLGAAERRKLAGKMQLSEVQIKTWFQNRRMKLKRQLQD

>Wallaby_Ventx/122-182

RRLRTAFTTKQISTLESSFKRHHYLGAAERRKLAGKMQLSEVQIKTWFQNRRMKLKRQLQD

>Opossum_Ventx2/108-168 LOC103102624 gi|612060062|ref|XP_007506754.1| PREDICTED: homeobox protein vent1-like [Monodelphis domestica]

RRLRTAFTTKQISTLESSFKRHHYLGAAERRKLAGKMQLSEVQIKTWFQNRRMKLKRQLQD

>King_Cobra_Ventx2/167-227

RRLRTAFSLEQISTLESSFKRHKYLGAAERRKLASKMQLSEVQIKTWFQNRRMKLKRQLQE

>Crotalus_Ventx2/151-211

RRLRTAFSLEQIGTLESSFKRHKYLGAAERRKLASKMQLSEVQIKTWFQNRRMKLKRQLQE

>Phyton_Ventx2/69-129

RRLRTAFSLEQISTLESSFKRHKYLGAAERRKLASKMQLSEVQIKTWFQNRRMKLKRQLQE

>Anole_Lizard_Ventx2.1/144-204

RRLRTAFSLEQISTLESAFQRHRYLGAAQRRKLAAKMRLSEVQIKTWFQNRRMKLKRQMQE

>Anole_Lizard_Ventx2.2/20-80

RRLRTAFSLEQISTLESAFQRHRYLGAAQRRKLAAKMRLSEVQIKTWFQNRRMKLKRQMQE

>Ara_Macao_Ventx2/97-157 s_1_1_contig94256, whole genome shotgun sequence

RRLRTAFSAEQVSTLESSFQRQQYLGAAERRKLAGRMRLSEVQIKTWFQNRRMKLKRQLQE

>Budgerigar_Ventx2/60-120

RRLRTAFSAEQVSTLESSFQRQQYLGAAERRKLAGRMQLSEVQIKTWFQNRRMKLKRQLQE

>Chinese_Turtle_Ventx2/160-220

RRLRTAFSVEQISTLESSFKRHKYLGSAERRKLAAKMQLSEVQIKTWFQNRRMKLKWQLQE

>Green_Turtle_Ventx2/109-169

RRLRTAFSVEQISTLESSFKRHKYLGSAERRKLAAKMQLSEVQIKTWFQNRRMKLKRQLQE

>Chicken_Ventx2/134-194

RRLRTAFSAEQISTLESSFQRHRYLGAAERRKLAGRMRLSEVQIKTWFQNRRMKLKRQLQE

>Crow_Ventx2/44-104 >gi|726992252|ref|XP_010408490.1| PREDICTED: homeobox protein VENTX [Corvus cornix cornix]

RRLRTAFSAEQISTLESSFQRQQYLGAAERRQLAGRMRLSEVQIKTWFQNRRMKLKRQLQE

>Xenopus_Laevis_Ventx.2.2/175-235

RRLRTAFTSDQISTLEKTFQKHRYLGASERQKLAAKLQLSEVQIKTWFQNRRMKYKREIQD

>Xenopus_Trop_Ventx2.1/190-250

RRLRTAFTSDQISTLEKTFQKHRYLGASERRKLAAKLQLSEVQIKTWFQNRRMKYKREIQD

>Xenopus_Laevis_Ventx2.1/185-245 Ventx2.1

RRLRTAFTSDQISTLEKTFQKHRYLGASERRKLAAKLQLSEVQIKTWFQNRRMKYKREIQD

>Platyfish_Vox/123-183

RRVRTKFTPEQISRLEKIFSKHKYLDAGERVKTAQKLNLTETQVRTWFQNRRMKLKREVQD

>Medaka_Vox/123-183 Ventx

RRVRTKFTPKQILKLEKVFSKHKYLDAGERVKTAQKLNLSETQVRTWFQNRRMKLKREVQD

>Tongue_Sole_Vox_/128-188 >gi|657786821|ref|XP_008320431.1| PREDICTED: homeobox protein pv.1-like [cynoglossus semilaevis ]

RRVRTKFTPEQINKLEKIFNKHKYLDAGERVKTAQRLNLTETQVRTWFQNRRMKMKREVQD

>Cod_Vox/121-181 Vox

RRLRTKFTPEQINKLEKIFNKHKYLDAGERLKTAQKLNLSETQIRTWFQNRRMKLKREVQE

>Coelacanth_Menadonis_Ventx/75-135 TSA: Latimeria menadoensis Latmen_c20573 transcribed RNA sequence

QRLRTAFTSEQIYNLEKTFKRHKYLGASERLKLAAKLQLSEIQIKTWFQNRRMKMKRQLQD

>Coelacanth_Ventx/115-175

QRLRTAFTSEQIYNLEKTFKRHKYLGASERLKLAAKLQLSEIQIKTWFQNRRMKMKRQLQD

>Spotted_Gar_Vox/141-201 Gar ENSGACP00000003761 (Vox)

RRIRTKFTSDQIYKLEKTFSKDKYLGATERLKLAAKLNLSEIQVKTWFQNRRMKLKREMQD

>Cod_Vent/59-119 Vent1

RRMRTKFTSEQINRLEDTFGRHKYLGATQRRKIAEKLSLSETQVKTWFQNRRMKLKRDLQD

>Astyanax_Mexicanus_Vent/104-164 LOC103039812 gi|597732752|ref|XP_007228709.1| PREDICTED: homeobox protein vent1-like [Astyanax mexicanus]

RRVRTKFTTYQISRLEKTFNKHKYLGATQRKKIAEKLHLSETQVKTWFQNRRMKLKREVQD

>Zebrafish_Vent/65-125 vent1

RRVRTKFTCDQISGLEKSFSKHRYLGATQRRKIAEKLHLSETQVKTWFQNRRMKLKREVQD

>Astyanax_Mexicanus_Vox/126-186 LOC103045048 gi|597732787|ref|XP_007228723.1| PREDICTED: homeobox protein vent1-like [Astyanax mexicanus]

RRVRTKFTPEQIDKLEKIFNKHKYLDAGERVKTALKLSLSETQVRTWFQNRRMKLKREVQE

>Tilapia_Vox/33-93 Oreochromis niloticus contig048699

RRVRTKFSPEQVKKLERIFIKQKYLDAGEREKTAQKLNLTETQVRTWFQNRRMKLKREVQD

>Neolamprologus_brichardi_Vox/33-93 Neolamprologus brichardi contig049650

RRVRTKFSSEQIKKLERIFIKQKYLDAGEREKTAQKLNLTETQVRTWFQNRRMKLKREVQD

>Maylandia_Zebra_Vox/33-93 Maylandia zebra contig021768

RRVRTKFSSEQIKKLERIFIKQKYLDSGEREKTAQKLNLTETQVRTWFQNRRMKLKREFQD

>Spotted_Gar_Ved/134-194

RRPRTAFTSEQISRLERTFKKHAYLGTREKEELCRKLNLSEKQIKNWFQNRRMKLKRTLQD

>Astyanax_Mexiucanus_Ved/49-109

RRARTAFTAEQIESLERAFKRNAYLGAQDKAELCKRLSLSDKQIRNWFQNRRMKLKRTVQD

>Zebrafish_Ved/141-201

RRPRTAFSSEQISSLERVFKRNAYLGAQDKAELCRTLKLTDKQIRNWFQNRRMKLKRTVQD

>Astatotilapia_burtoni_Vent/1-61 Haplochromis burtoni contig023015

RRLRTKFTSEQVSKLEHTFSKQKYLGATQRRKIAEELNLSETQVKTWFQNRRMKLKREVQD

>Astatotilapia_burtoni_Vox/33-93 Haplochromis burtoni contig023015

RRVRTKFSSEQIKKLERIFIKQKYLDSGEREKTAQKLNLTETQVRTWFQNRRMKLKREFQD

>Pundamilia_Nyererei_Vox/33-93 Pundamilia nyererei contig020489

RRVRTKFSSEQIKKLERIFIKQKYLDSGEREKTAQKLNLTETQVRTWFQNRRMKLKREFQD

>Stegastes_partitus_Vox/127-187 gi|657562942|ref|XP_008284891.1| PREDICTED: homeobox protein vent1-like [Stegastes partitus] LOC103360771

RRVRTKFTAEQINKLEKIFNKHKYLDAGERVKTAQKLNLTETQVRTWFQNRRMKLKRELQD

>Chinese_Turtle_Ventx1/119-179 turle Vent1 ENSPSIP00000004010

RRARTAFTPEQVGRLERTFQRQRYLGAAERRKLATALHLSEIQVKTWFQNRRMKFKRQMQD

>Zebrafish_Vox/141-201 Vox

RRIRTKFTPEQIDKLEKIFNKHKYLDAGERVKTALKLGLSETQIRTWFQNRRMKLKREVQE

>Anguilla_Japonica_Vox/138-198 contig_2724, whole genome shotgun sequence

RRARTKFTSEQIYKLEKTFKKHKYLDPTERIKTAEKLNLSETQVRTWFQNRRMKLKRDVQD

>Little_Skate_VentxA/181-241 Leucoraja erinacea LER_WGS_1_CONTIG_1613628

RRVRTAFTAQQIHKLEKKFKRQTYLGASERSKLAALLHLSETQVKTWFQNRRMKVKRIVKD

>Elephant_Shark_VentxA1/135-195 SINCAMP00000019661

RRARTAFTAQQIHRLEKRFKRQTYLGGSERVRLAASLHLSETQVKTWFQNRRMKLKRQLQD

>Elephant_Shark_VentxA2/123-183 SINCAMG00000012859

RRARTAFTAQQIHRLEKKFTHQKYLGASERVRLAASLHLSETQVKTWFQNRRMKLKRQLQD

>Elephant_Shark_VentxB/139-199 SINCAMP00000019665

RRPRTIFSVQQLSILETSFQHQPYPGTCQRQRLAGALSLSETQVKTWFQNRRMKLKQQLQD

>Little_Skate_VentxB/39-99 Skate Vx1

RRLRTIFTVEQVRSLEFSFQRQQYPGSCVRRTLAGELRLSEAQVKTWFQNRRMKLKQQLQV

>Anguilla_Japonica_Vent/1-61 contig_2726, whole genome shotgun sequence

RRMRTKFTSDQIYRLEKTFNKHKYLGATQRRKMAERLHLSETQVKTWFQNRRMKLKREVQD

>Coelacanth_Ved/162-222

RRPRTAFTVEQINKLEKTFNKQKYLASQERQELCRKLNLSEKQIKTWFQNRRMKLKRNIQD

>Tilapia_Vent/1-61 Oreochromis niloticus contig048699

RRLRTKFTSEQVSKLEHTFSKQKYLGATQRRKIAEELNLSETQVKTWFQNRRMKLKREVQD

>Maylandia_Zebra_Vent/5-65 Maylandia zebra contig021767

RRLRTKFTSEQVSKLEHTFSKQKYLGATQRRKIAEELNLSETQVKTWFQNRRMKLKREVQD

>Pundamilia_Nyererei_Vent/1-61 Pundamilia nyererei contig020489

RRLRTKFTSEQVSKLEHTFSKQKYLGATQRRKIAEELNLSETQVKTWFQNRRMKLKREVQD

>Neolampromogus_bichardi_Vent/1-61 Neolamprologus brichardi contig049650

RRLRTKFTSEQVSKLEHTFSNQKYLGATQRRKIAEELNLSETQVKTWFQNRRMKLKREVQD

>Collared_Flycatcher_Ventx2/1-61

RRLRTAFSAEQISTLESSFQRQQYLGAAERRQLAGRMRLSEVQIKTWFQNRRMKLKRQLQE

>Painted_Turtle_Ventx2/150-210

RRLRTAFSVEQISTLESSFKRHKYLGSAERRKLAAKMQLSEVQIKTWFQNRRMKLKRQLQE

>Manatee_Ventx/144-204

PRVRTAFTTAQISTLESAFKLRQYLGPQERKKLAKEMQLTEVQIKTWFQNRRMKHKRQMQD

>Lesser_Hedgehog_Tenrec_Ventx/73-133 Echinops telfairi contig066530

PRLRTAFTAVQLSTLESAFQLHQYLGPQERKTLAQEMQLTEMQIKTWFQNRRMKHKRQMQD

>Sloth_Ventx/11-71 ENSCHOP00000009883 Ventx

PRVRTAFTAAQVSTLESSFQHRRYLGPPERKKLAEELQLSEVQIKTWFQNRRMKHKRQMQD

>Weddell_Seal-Ventx/13-73

PRVRTAFTAEQVSTLESAFQHHRYLGPlERRRLARELRLSEVQVKTWFQNRRMKHKRQLQD

>Sperm_Whale_Ventx/19-79

PRVRTASTAEQVSALEYSFRHRRYLGPLEHRRLAREMRLSEVQLLTWFQNRRVKYKRHVQG

>Puerto_Rican_Parrot_Ventx2/1-61

RRLRTTFSAEQVSTLESSFQRQPYLGAAERRKLSGRMRLSEVQIKTWFQNRRMKLKRQLQE

>Duck_Ventx1/4-64

RRARTAFTAEQVCRLEKTFQRQKYLGASERRKLAAALQLSEIQIKTWFQNRRMKLKRQIQD

>ZfinchVentx3/57-117

GRPRTKFSAAQLQELERSFREQRYIGAGEKRRLAAVLNLSQSQIKTWFQNRRMKFKRQTQD

>Axolotl_Ventx1/56-116

RRARTAFSSQQLSRLEQTFAKQQYLPAQERRKLATALQISEMQIKTWFQNRRTKMKRQTLD

>Pseudacris_Ventx1/2-62

KRIRTAFSPSQLLRLERAFEKNHYVVGAERKQLASSLSLSETQVKVWFQNRRTKYKRQKLE

>Zonotrichia_Albicollis_Ventx1/120-180 PREDICTED: homeobox protein vent1-like [Zonotrichia albicollis] gi|542145232|ref|XP_005481664.1|

RRARTAFTSEQVCRLEKTFQRHKYLGATERRKLAAALQLSEIQIKTWFQNRRMKLKRQIQD

>Alligator_Mississippiensis_Ventx1/42-102 VXXX

RRLRTAFTSTQICQLEKTFKRQKYLGAAERRKLAAALQLSEVQVKTWFQNRRMKLKRQIQD

>Painted_Turtle_Ventx1/1-60 Chrysemys picta bellii Contig138.15

-RARTAFTPEQVGKLEKTFKRQKYVGAAERRKLAAALQLSEIQVKTWFQNRRMKLKRQIQD

>Platypus_Ventx1/153-213 LOC100076658 Ventx

RRARTAFSPAQVCLLEKTFKGQRYLGIGDRKKLATNLNLSEVQVKTWFQNRRMKLKRQLQD

>Alligator_Sinensis_Ventx1/1-61

RRLRTAFTSTQICQLEKTFKRQKYLGAAERRKLAAALQLSEVQVKTWFQNRRMKLKRQIQD

>Green_Turle_Ventx1/14-74

RRARTAFTPEQVGKLEKTFKRQKYVGAAERKKLAAALQLSEIQVKTWFQNRRMKLKRQIQD

>Xenopus_Ventx3.1/123-183

SRARTKFTPEQLKELERSFKENMYIGSSEKRRLSKVLKLSECQIKTWFQNRRMKLKRQSQD

>RanaVentx3/123-183

SRPRTKFSDEQLRELETSFREQKYIGSNEKKRLSRMLNLSETQIKTWFQNRRMKFKRQNQD

>PseudacrisVentx3/168-228

SRPRTKFTAEQLQELEKSFKEHRYIGSSEKKRLSKVLKLSETQIKTWFQNRRMKFKRQSQD

>Axolotl_Ventx3/50-109 Ventxx

-RPRTKFSTEQLQELERSFQEQRYIGVAEKRRLARELNLSELRIKTWFQNRRMKFKRQNQD

>Pseudopodoces_humilis_Ventx3/101-161 XP_005520905

GRSRTKFSAAQLQELERSFREQRYIGAGEKRRLAAVLNLSQSQIKTWFQNRRMKFKRQTQD

>Chicken_Ventx3/103-163 XP_004942219

ARPRTKFSAVQLQELERSFREQRYIGASEKRRLAAALDLSQSQIKTWFQNRRMKFKRETQD

>Painted_Tutle_Ventx3/1-60 Chrysemys picta bellii Contig138.15

-RPRTKFSASQLQELERSFREQRYIGASEKRRLSKVLKLSQTQIKTWFQNRRMKFKRQTQD

>Alligator_Sinensis_Ventx3/1-61

GRPRTKFSASQLQELERSFQEQRYIGASEKRRLSKVLNLSQTQIKTWFQNRRMKFKRQTQD

>Budgerigar_Ventx3/1-60

-RPRTKFSAAQLQELERSFREQRYIGTSEKRRLAAVLNLSQGQVKTWFQNRRMKFKRQTQD

>Budgerigar_Ventx1/117-177 XP_005144153

RRARTAFTSDQVCRLEKTFQRQKYLGASERRKLSAALQLSEIQIKTWFQNRRMKLKRQIQD

>XenopusVentx1.1/132-192 Ventx1.1

RRLRTAFTPQQISRLEQAFNKQRYLGASERKKLATSLRLSEIQVKTWFQNRRMKLKRQIQD

>Alligator_Mississippiensis_Ventx3/1-60 Ventx3

-RPRTKFSTSQLQELERSFQEQRYIGASEKRRLSKVLNLSQTQIKTWFQNRRMKFKRQTQD

>Coelacanth_Ved/16-76 Ved

RRPRTAFTVEQINKLEKTFNKQKYLASQERQELCRKLNLSEKQIKTWFQNRRMKLKRNIQD

>Anguilla_japonica_Ved/1-61 Anguilla japonica contig_301331

RRPRTAFTAEQISRLEKAFKRNAYLGTHDKAELCKQLSLSDKQIRNWFQNRRMKLKRTLQD

>Cod_Ved/4-64

RRPRTAFTAEQVHSLERAFKKNAYLGTQDKSELCRKLNLSDKQIRNWFQNRRMKLKRTVQD

>Medaka_Ved/1-61 Oryzias latipes DNA, contig84 in scaffold1

RRPRTAFTAEQIDVLERAFKKNAYLGTQDKAELCRKLRLSDKQIRNWFQNRRMKLKRSVQD

>Tilapia_Ved/1-61

RRPRTAFTAEQISSLEKAFSISAYLGTQSKAELRKKLHLSDKQIRNWFQNRRMKVKRTMQD

>Cod_Ved/2-62 Ved

RRPRTAFTAEQVHSLERAFKKNAYLGTQDKSELCRKLNLSDKQIRNWFQNRRMKLKRTVQG

>platyfishII/126-186

RRPRTAFTAEQISSLEGAFKRNAYLGTQDKAELCKKLNLSDKQIRNWFQNRRMKLKRTVQD

>Saccoglossus_Vent1/108-168 Ventx1

KKARTAFTNEQIGLLEKRFRLQKYLSATERVEFAESIGLTDTQVKTWFQNRRMKWKRQKKD

>Saccoglossus2_Vent2/69-129 VENTX2

KRARTAFSNEQVYKLEKRFRAQKYLSATEREDVSRSIGLSDTQVKTWFQNRRMKWKRERKD

>AmphiVent1/117-177 Jgi Sc.777 Protein ID:290585

RKARTAFTTEQVMALEERFRLQKYLSAADRETLAKATGLTDEQVKTWFQNRRMKLKRQQQD

>AmphiVent1b/117-177 Jgi Sc. 185

RKARTAFTTEQVMALEERFRLQKYLSAADRETLAKATGLTDEQVKTWFQNRRMKLKRQQQD

>AmphiVent2a/114-174 Jgi Sc.777 Protein ID:289443

RKARTAFTTEQVMALEERFRLQKYLSAADRETLAKATGLTDEQVKTWFQNRRMKLKRQQQD

>Amphivent2b/69-129 sc.185

RKARTAFTTEQVMALEERFRLQKYLSAADRETLAKATGLTDEQVKTWFQNRRMKLKRQQQD

>AmphiLcx1/143-203 Jgi Sc.777 Protein ID:290453

KKLRTAFSSHQVHELETRFSTQKYLSASDREELSQALDLTDAQVKTWFQNRRMKWKRQVQD

>AmphiLcx2/143-203 Sc.185

KKLRTAFSSHQVHELETRFSTQKYLSASDREELAHALDLTDAQVKTWFQNRRMKWKRQVQD
